# Data for the stress update assessment in large-deformation finite element analysis

**DOI:** 10.1016/j.dib.2023.109994

**Published:** 2023-12-21

**Authors:** Fabrizio Antonio Stefani, Ramon Francesconi

**Affiliations:** Department of Mechanical Engineering, Energetics, Management and Transportation (DIME), Polytechnic School - University of Genoa, Via Opera Pia, 15A, Genoa 16145, Italy

**Keywords:** Finite element analysis, Geometrical non-linearity, Large displacement, Stress update, Objectivity, Work conjugacy

## Abstract

During a research work about stress integration schemes for large-deformation finite element analysis many datasets are collected from both numerical and analytical models. The corresponding numerical data (stress and displacements) are computed by means of different finite element formulations including the well-established stress update schemes employed by the major commercial software packages. To this purpose a suitable finite element code, capable of easily switching the different methods, is implemented. Accordingly, the data computed for three stress integration tests allowing analytical solution in the case of linear material are presented. The comparison of all the predictions from the various methods allows the choice of the most accurate model in predicting displacement and related stress. In addition, the data may be reused as starting point in the development of new stress integration strategies, as a reference comparison to understand the behaviour of the standard methods.

Specifications Table

**Table:** 

Subject	Computational Mechanics
Specific subject area	Finite element structural analysis (simulation of geometric nonlinearity due to large displacements)
Data format	Raw, Analyzed and/or Filtered
Type of data	Table, Figure included in .xlsx files (dataset with numbers and plots) .ans file (APDL script)
Data collection	The data have been computed by means of in-house developed FE software (a code referred to as FEMLub), capable of emulating the stress-update behaviour of four categories of commercial codes, as well as the software package Ansys (version 2021 R1).
Data source location	Institution: University of Genoa City/Town/Region: Genoa Country: Italy Latitude: 44.414165, longitude: 8.942184.
Data accessibility	Repository name: Mendeley Data Data identification number: Version 3, DOI: 10.17632/mx3nj27ddh.3 Direct URL to data: https://data.mendeley.com/datasets/mx3nj27ddh/3
Related research article	F. Stefani, M. Frascio, C. A. Niccolini Marmont Du Haut Champ, Choice of the stress integration scheme for accurate large-deformation finite element analysis, Proc IMechE Part C: J Mechanical Engineering Science, 2023, Vol. 237(17) 3977–3986

## 1 Value of the Data

• The comparison between numerical and theoretical data of stress integration tests allows the assessment of the performance of different well-established stress integration methods employed in commercial FE codes.
• All the FE analysts among the mechanical designers can benefit from these data, which can be used to properly choose the stress integration method or the commercial code for their structural simulations inclusive of geometrical non-linearity depending on the strain conditions to be analyzed.
• Since the choice of stress update procedure is distinctive in the development of FE codes for large-displacement analysis, the data can be reused by researchers for assessment and enhancement of their accuracy. The comparison may be extended by other researchers to other important stress update methods.
• By means of the comparison with the exact solutions, the numerical data can be analyzed to identify and explain possible inaccuracies of each stress update method. Although in the related research theoretical interpretation of the main inaccuracy is given, understanding the sources of the inconsistencies requires further work.

## 2 Objective

The goal for creating the dataset was to explain the different predictions of popular commercial software even in a simple example of large-displacement analysis with linear material, where large differences (up to 60%) in the main output variables have been found among different codes [1]. The assessment of the stress-update procedure is essential to finding an explanation for such evidence, as the related research has proved that the choice of stress update procedure is distinctive in the development of a FE code for large-displacement analysis, while the remaining algorithmic differences between the analyzed codes are marginal.

The dataset [2] allows us to assess each stress integration method by comparing its response with the exact theory. It includes only the stress integration methods used in commercial software and, for further research, requires an extension to more complex methods like those exploiting the logarithmic stress rate proper to design an “exact” stress update formulation [3], as well as those suitable to non-linear (e.g., elastoplastic) materials, for which the procedures require appropriate adaption [4].

The purpose of sharing the dataset is to enable other analysts and researchers to interpret and understand the nature of the disagreements of the reported as well as possible additional data. As an example of utility of the dataset, the related research work has already identified and theoretically explained a systematic inaccuracy of some stress update methods. Such an issue causes a large numerical error when traction loading conditions are involved, i.e., in almost all the static structural simulations including large deflection computation. Indeed, the benchmark problem of traction strain presented in [1] provides the evidence of relative variations in numerical predictions among the different methods equal to 65.5 % and 35.2 % (with reference to the “true” results) for maximum displacement and Von Mises stress, respectively.

## 3 Data Description

The data reported in the present article as well as in repository [2] include the exact (analytical) solutions of four stress integration tests and the corresponding numerical predictions computed by different stress-update methods.

**Fig. 1:**
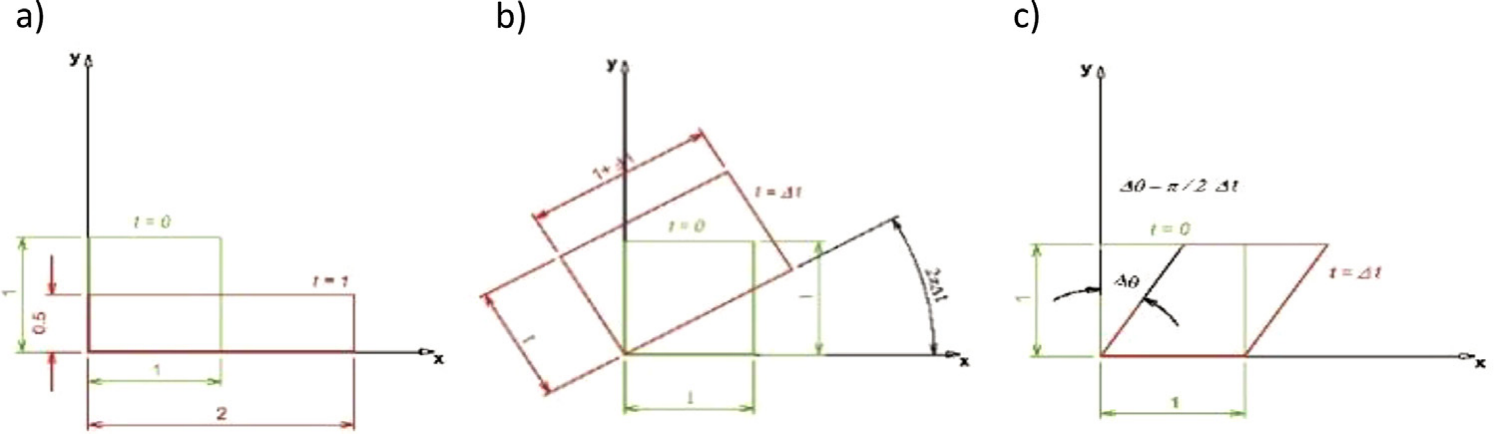
Schemes of stress integration tests: a) “extension-compression”; b) “extension-rotation”; c) “simple shear”.

In the stress integration tests a unit square (1×1 m^2^) with unit thickness undergoes a deformation path under plane stress conditions. It is made of linear elastic material with Young's modulus E = 1 Pa and Poisson ratio ν = 0. Specific testing conditions are extracted from different papers, i.e., [5], [6], [7], [8], [9]. Accordingly, the stress integration algorithms are checked by means of three tests: “extension-compression”, “extension-rotation”, “simple shear”. These three paths are summarized in Fig. 1 and include the most common motions of the structure particles (strain and rotations). The “uniaxial extension” path, which defines a stretch in x direction, has been added afterwards, as its inherent simplicity has allowed the in-depth study of a systematically inaccurate numerical behaviour spotted in the “extension-compression” case. A fixed number of time steps N equal to 50 is chosen. The simulated “time” parameter t ranges between 0 and 1 in “uniaxial extension”, “extension-compression”, “extension-rotation” tests, whereas it raises from 0 to 0.9 in “simple shear” test. Therefore, the corresponding time steps Δt are equal to 1/50 and 9/500, respectively.

For compression-extension, extension-rotation, simple shear, and uniaxial extension test case the relevant raw data (stress predictions) are respectively gathered in the following four files together with the corresponding plots: *results_comp_ext_repository.xlsx, results_ext_rot_repository.xlsx, results_simple_shear_repository.xlsx, results_uniaxial_extension_repository.xlsx*.

The remaining Excel spreadsheet file *cc_methods_validation_repository.xlsx* gathers some raw data (stress time-histories as in the files mentioned above) computed for the “simple shear” test in order to validate the Corotated Configuration stress-update algorithms (see the section “Methods”) developed in the related research work and to verify their implementation. To this purpose, the stress components computed numerically are compared with their theoretical counterparts calculated analytically according to exact, Zaremba-Jaumann and Green-Naghdi theory.

The APDL script *test_Rodriguez.ans* collects the commands required to calculate the numerical data for the stress-update methods employed in Ansys commercial software, i.e., in the formulation of PLANE42 and PLANE182 elements. The same algorithm for reproducing the different stress integration tests has been exploited to develop both the above-mentioned script and a suitable C++ program. They are capable of checking the stress-update procedures employed in Ansys and those proposed in literature, respectively.

## 4 Experimental Design, Materials and Methods

The stress integration algorithms for Updated Lagrange (UL) large-displacement analysis considered hereinafter and in the research article [1] are divided into two groups: classic methods and those based on a Corotated Configuration (CC methods).

The methods in such groups, already cited and/or described in [1], are only briefly reminded in the related sub-paragraphs that follow. Differently, the remaining and more specific algorithms, implemented to validate CC methods and to check commercial software response, are more extensively explained in the corresponding sub-paragraphs, where some details about CC methods required to understand the context are also repeated.

### 4.1 Classic methods

Classic methods include: “Bathe linear”, “Bathe”, “Rodriguez 1”, “Rodriguez 2”, “Hughes-Winget”, “Gadala-Wang”, “Gadala-Wang nonlinear”, “Pinsky”.

“Bathe linear”, the simplest stress integration algorithm, relies on the forward Euler method where the stress increment is computed from linearized strain [10]. In “Bathe” method the Euler scheme prediction at each step is enhanced in that the strain linearization is avoided [11].

The “Hughes-Winget” method, by decomposing the deformation, computes the updated stress by adding a transformation (rotation) of current stress due to rigid body motion and a stress increment due to straining. The rotation tensor is assessed by using Jaumann objective stress rate as well as a mid-time step rule, in order to preserve the incremental objectivity of the integration procedure [12].

The same type of decomposition is assumed by the “Gadala-Wang” method, where the transformation is computed by means of the Truesdell stress rate and the strain is linearized to assess the stress increment [13]. Differently, “Gadala-Wang nonlinear” method employs the whole Green-Lagrange strain increment.

Among the explicit schemes in the family of transformations reported in [14] “Pinsky” method denotes the explicit integration technique related to Truesdell stress rate and applied to both current stress and stress increment, while “Rodriguez 1” and “Rodriguez 2” methods capture the inherent non-linear behaviour of large displacements in two different ways.

Specifically, “Rodriguez 1” method evaluates the stress increment by including quadratic terms in Green-Lagrange strain, whereas “Rodriguez 2” algorithm exploits a mid-time step rule reminiscent of “Hughes-Winget” method together with the stress increment linearization [6].

### 4.2 CC methods

CC methods are: “Material Green-Naghdi”, “Spatial Green-Naghdi”, “Mid-step Green-Naghdi”, “Hughes-Green-Naghdi”, “Hughes-Zaremba-Jaumann”.

According to the general definition of CC methods, all these variants use a rotation-neutralized configuration that co-rotates with the body so that it is not affected by relative rigid motions. The related quantities are suitably transformed in such configuration where the constitutive equation is integrated, and transformations are also used to rotate back the results in the updated configuration. In the reference research work [1] all the analyzed methods have been adapted to the UL formulation by adopting the basic assumption that the current spatial frame is used as rotation-neutralized configuration. The different variants of CC methods are characterized by the choice of the transformations and the computation of the deformation gradient, which is required as input by the algorithms.

“Hughes-Green-Naghdi” and “Hughes-Zaremba-Jaumann” schemes denote the CC methods developed by Hughes [15] by using corotational Green-Naghdi and Zaremba-Jaumann objective stress rates, respectively. Due to the above-mentioned basic assumption of CC methods for UL formulation the resulting transformations used in “Hughes-Green-Naghdi” and “Hughes-Zaremba-Jaumann” algorithms differ from the general corresponding relationships found in [8] in that the incremental rotations are used instead of the total ones. Both algorithms exploit interpolation to assess the total deformation gradient at the middle time step.

The remaining variants of CC methods are devised by altering the deformation gradient computation. In the “spatial Green-Naghdi” algorithm the increment of deformation gradient between current and middle time step is found from the incremental displacement derivatives evaluated in the spatial reference system (the current configuration). The “material Green-Naghdi” variant computes the total deformation gradient at middle time step by means of numerical differentiation of the displacements in the material reference system (the initial time configuration). In “mid-step Green-Naghdi” method the mid-time step configuration becomes the reference system for the computation of the corresponding incremental deformation gradient. The comparison of data in the repository [2] confirms that “Hughes-Green-Naghdi”, “spatial Green-Naghdi” and “material Green-Naghdi” algorithms are different sequences of operations that implements the same method, as theoretically predictable, while “mid-step Green-Naghdi” is a different procedure.

### 4.3 CC methods for commercial software emulation

By means of a benchmark problem the related research paper has compared well-established stress-update algorithms employed in four categories of commercial codes [1]. Specifically, software of the first category employs the Jaumann rate of the Cauchy stress incorrectly matched with constant material elasticity tensor; the codes in the second category are based on Green-Naghdi formulation; in the third category the Truesdell rate of Cauchy stress is adopted; more generically, codes in the fourth category exploit a CC method.

The data for the four stress integration tests have been collected by means of both FEMLub, a in-house FE code capable of easily switching the different stress-update methods, and a popular commercial software in the second category, i.e. the commercial software Ansys (version 2021 R1). The comparison with results from Ansys software has been used to verify some well-known features typical of Green-Naghdi formulation, e.g., the underestimation of tangential stress in “simple shear” test [5] (see Fig. 9(b) and Fig. 12(b)), and thus to check the algorithmic consistency in the implementation of the stress integration tests.

To this goal, the two additional CC methods referred to as “material Green-Naghdi (total rotation)” and “spatial Green-Nagdi (full rotation increment)” have been implemented according to the general description of Ansys theoretical manual [16] (at our best) in order to emulate the stress-update of PLANE42 and PLANE182 elements of the Ansys code, respectively. Particularly, those two elements respectively implement the legacy and current technology for plane element analysis in Ansys software.

As reported in the previous sub-paragraph, the basic hypothesis about the neutralized configuration is also retained in case of the “material Green-Naghdi” method, where the corresponding denomination is due to the computation of the total deformation gradient. Indeed, it has been derived by resorting to shape function derivatives in the material reference system, i.e., the initial time (*t* = 0) configuration. Differently, by assuming that the material frame is the rotation-neutralized configuration and substituting total rotations for the corresponding incremental ones in agreement with [8,16], a variant of “material Green-Naghdi” algorithm has been implemented and denoted by the suffix “total rotation” (in brackets) appended to the label as in Fig. 10, Fig. 11, Fig. 12.

The basic assumption above about neutralized configuration is also employed in the “spatial Green-Naghdi” method, which differs from “Hughes-Green-Naghdi” in that, instead of computing the total deformation gradient at half time step by interpolation as advised in [8], it is found in incremental form by summing half of the incremental displacement gradient computed in the current (spatial) reference and the identical matrix.

If in the computation of the spatial logarithmic strain the increment of the right stretch tensor between current and mid-time step time is replaced with its full increment during the time step as in the corresponding equation found in [16], a variant of the “spatial Green-Naghdi” method is obtained and it is referred to by appending the suffix “full rotation increment” (in brackets) to the relevant label as in Figs. 10–12.

### 4.4 CC methods used for validation

Since the algorithms used to implement the CC methods described in the reference paper [1], specifically designed for the UL formulation, are somehow different from those described in [8] as explained in the “CC methods” sub-paragraph, validation is required. To this goal, the analytical solutions of “Zaremba-Jaumann theory” and “Green-Naghdi theory” for the simple shear test reported in [8], are used as reference. The raw data of both analytical and numerical models are collected in the file *cc_methods_validation_repository.xlsx*.

Particularly, although “Zaremba-Jaumann theory” employs a stress rate that behaves like a work-conjugated one by means of a proper correction of the elasticity tensor, it assumes a constant material elasticity tensor and, therefore, does not fulfil work-conjugacy requirement, i.e., second order accuracy of internal work is not preserved. In order to compare the corresponding numerical approach with such theoretical model, the “Hughes-Zaremba-Jaumann” integration method has been modified by using constant elasticity tensor as in “Zaremba-Jaumann theory”. The resulting numerical method is labelled by appending the “constant C” suffix (in brackets) to the label of such method (see Fig. 5).

Differently, in order to validate the CC methods that use Green-Naghdi stress rate, both “Zaremba-Jaumann theory” and “Green-Naghdi theory” can be used. Indeed, as explained in detail in [8], the Green-Naghdi stress rate reduces to the Zaremba-Jaumann one, if the current configuration is taken as reference for the total displacement gradients. Such assumption is identified by means of the suffix “F=I” (in brackets) appended to the label of the modified method, as reported in the legend of Fig. 5.

## 5 Data Comparison

The comparisons of data from each category of methods are included in each file of the repository [2] in graphical form. In order to illustrate the content of the dataset, in the following the most significant of these plots are also reported and commented on. Their interpretation (not topic of the present paper) is trivial by taking as reference the analytical solutions and it is supported by the related research paper [1] (paragraph “Stress integration tests”). The comparisons of the trends are presented according to the classification of the computational methods (classic, CC, for software emulation).

### 5.1 Comparison of classic stress integration test data

**Fig. 2:**
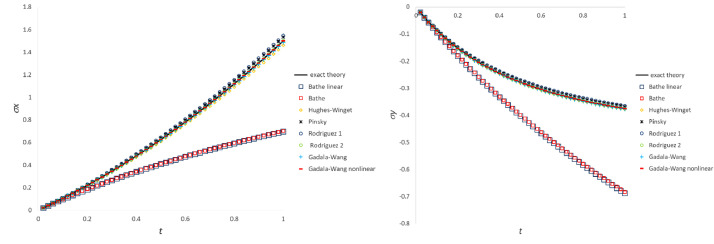
Stress history predicted by classic UL algorithms for extension-compression test: a) σ_x_ component; b) σ_y_ component.

**Fig. 3:**
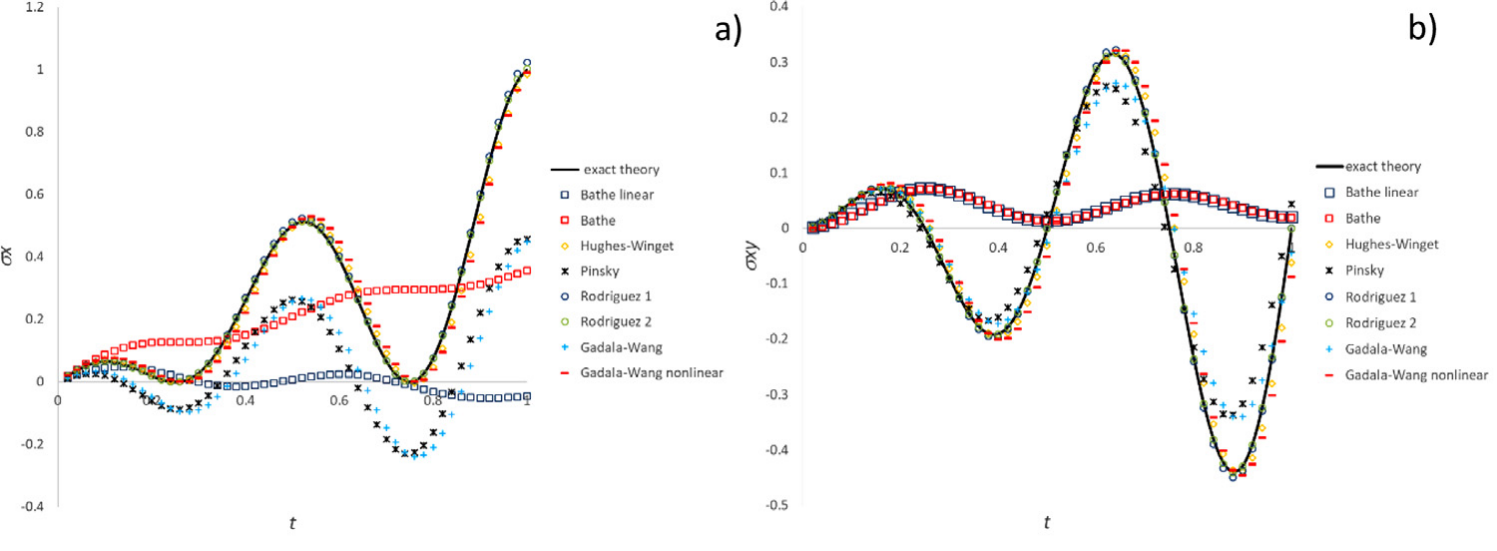
Stress history predicted by classic UL algorithms for extension-rotation test: a) σ_x_ component; b) σ_xy_ component.

**Fig. 4:**
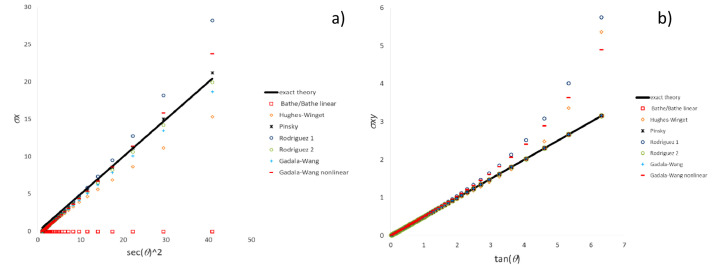
Stress history predicted by classic UL algorithms for simple shear test: a) σ_x_ component; b) σ_xy_ component.

The present section reports the data obtained in the three tests including the most common particle motions by means of the classic stress integration methods. For the compression-extension test, Fig. 2(a) and (b) plot the time-histories of stress components σ_x_ and σ_y_, respectively (σ_xy_ is zero for all the methods in agreement with theory). Time-histories of stress components σ_x_ and σ_xy_ due to extension-rotation test are plotted in Fig. 3(a) and (b), respectively. Since agreement with the “exact theory” of σ_x_ and σ_y_ component is similar, the plot of the history of stress σ_y_ included in the repository file *results_comp_ext_repository.xlsx* is not reported here. In the case of simple shear test Fig. 4(a) and (b) show trends of stress components σ_x_ and σ_xy_ against square secant and tangent of distortion angle θ defined in Fig. 1, respectively.

### 5.2 Comparison of CC stress integration test data

**Fig. 5:**
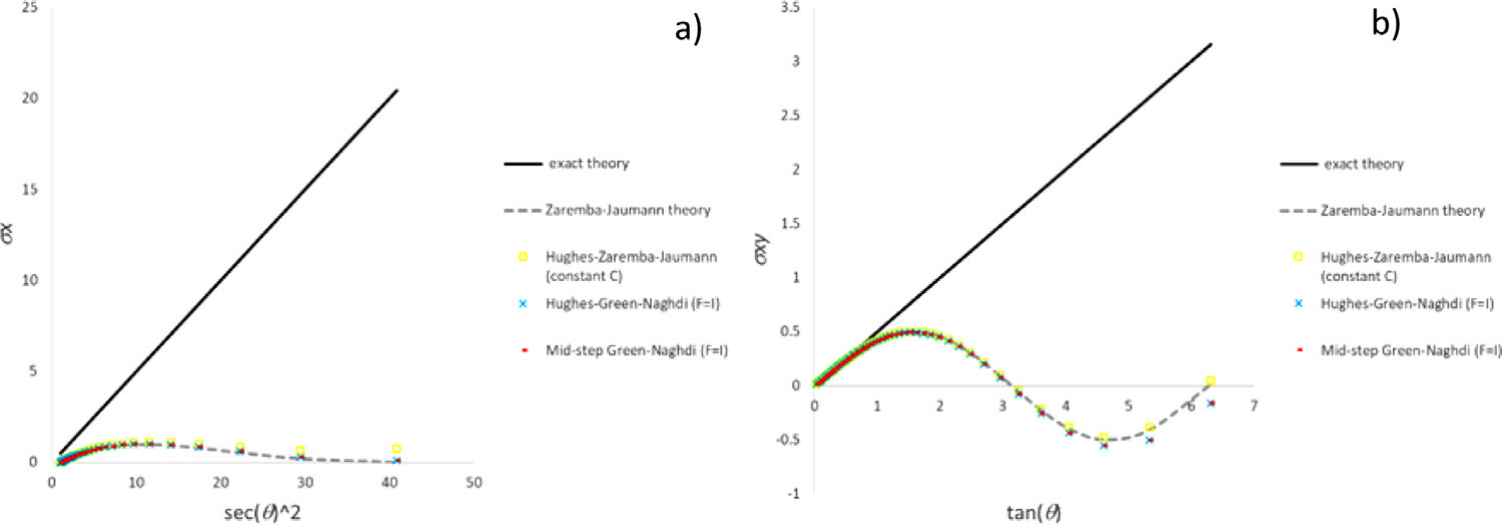
Validation of CC integration methods by comparison of stress history with Zaremba-Jaumann theory for simple shear test: a) σ_x_ component; b) σ_xy_ component.

**Fig. 6:**
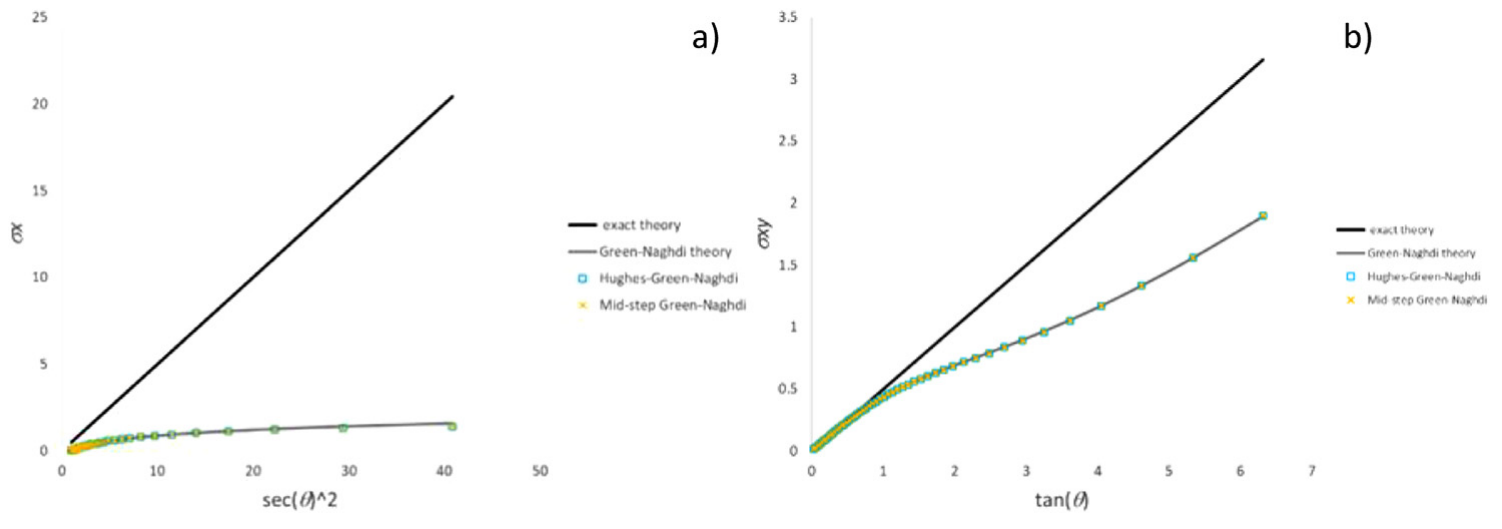
Validation of CC integration methods by comparison of stress history with Green-Naghdi theory for simple shear test: a) σ_x_ component; b) σ_xy_ component.

**Fig. 7:**
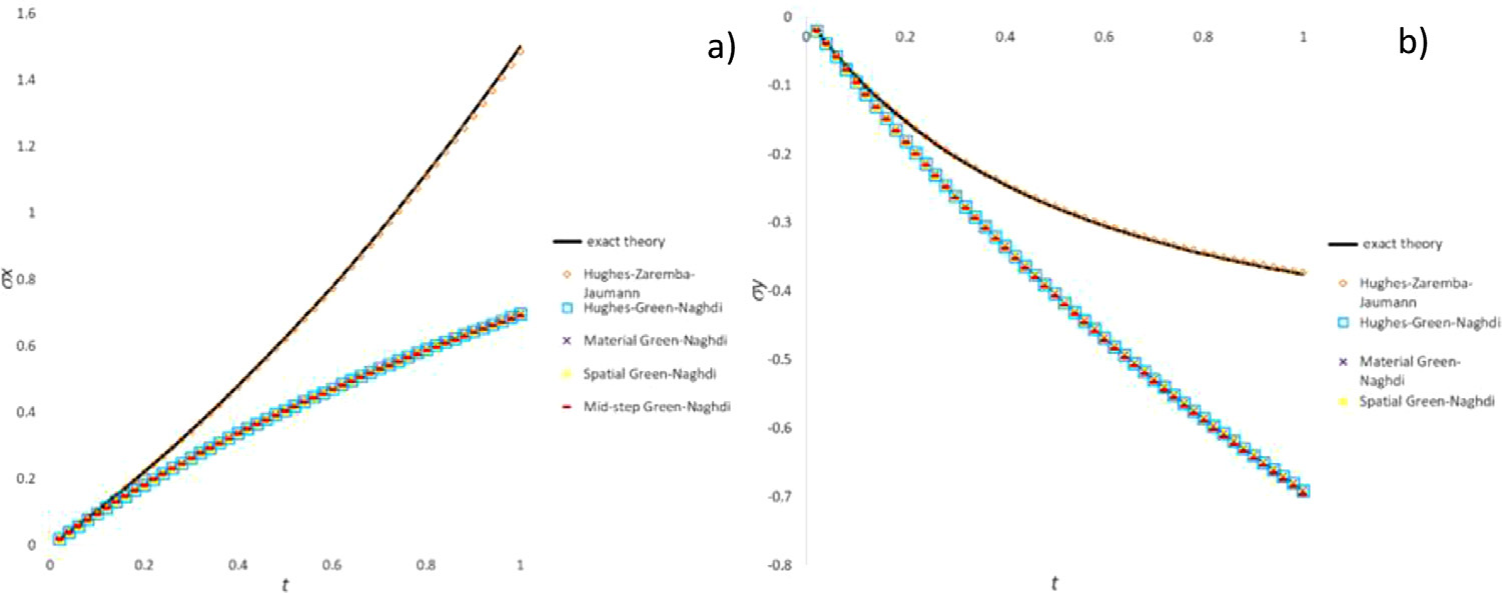
Stress history predicted by CC algorithms for extension-compression test: a) σ_x_ component; b) σ_y_ component.

**Fig. 8:**
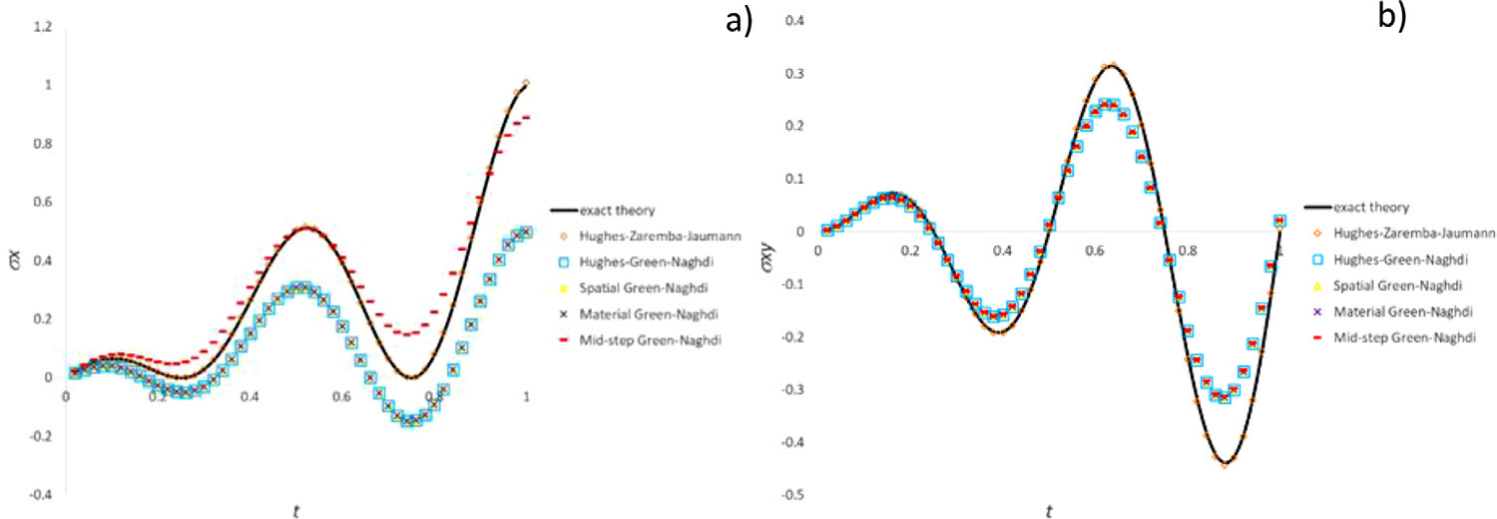
Stress history predicted by CC algorithms for extension-rotation test: a) σ_x_ component; b) σ_xy_ component.

**Fig. 9:**
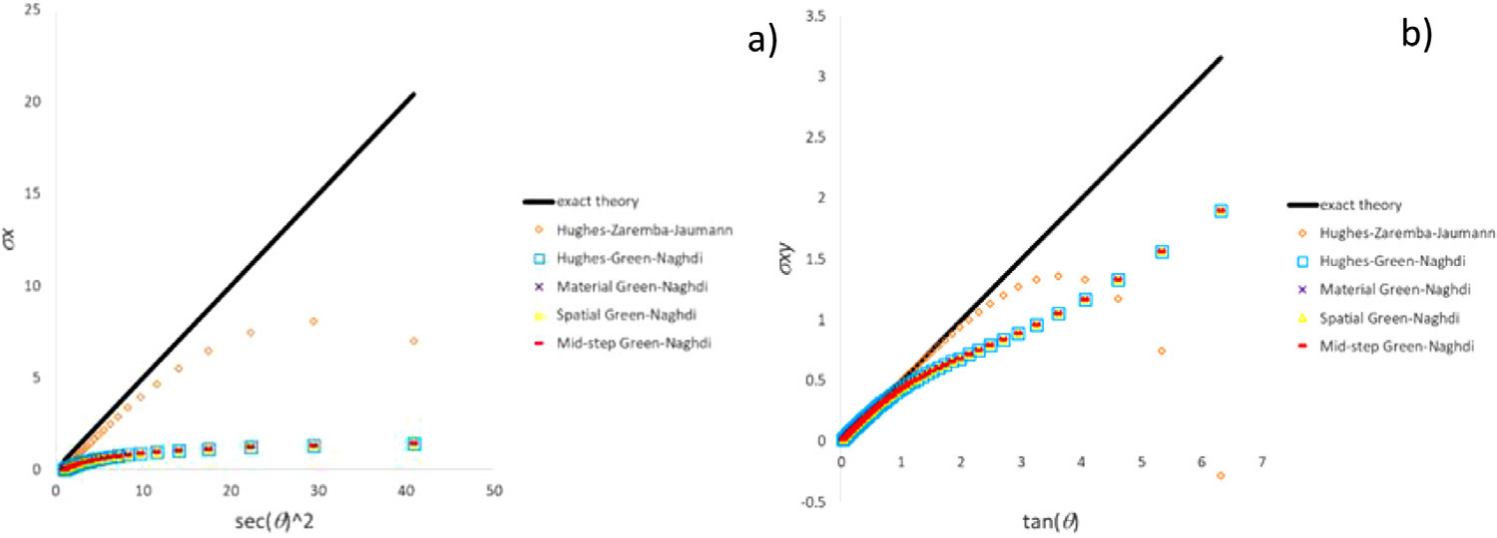
Stress history predicted by CC algorithms for simple shear test: a) σ_x_ component; b) σ_xy_ component.

Fig. 5 validates the stress component trends calculated for the simple shear test by means of both “Hughes-Zaremba-Jaumann (constant C)”, “Hughes-Green-Naghdi (F=I)” and “mid-step Green-Naghdi (F=I)” methods against “Zaremba-Jaumann theory”. Likewise, Fig. 6 validates “Hughes-Green-Naghdi” and “mid-step Green-Naghdi” methods against “Green-Naghdi theory” for the simple shear test. Fig. 7, Fig. 8, Fig. 9 compare in the three test cases including the most common particle motions the histories of the pertinent stress components computed by means of CC methods in each category.

### 5.3 Comparison of methods for commercial software emulation

**Fig. 10:**
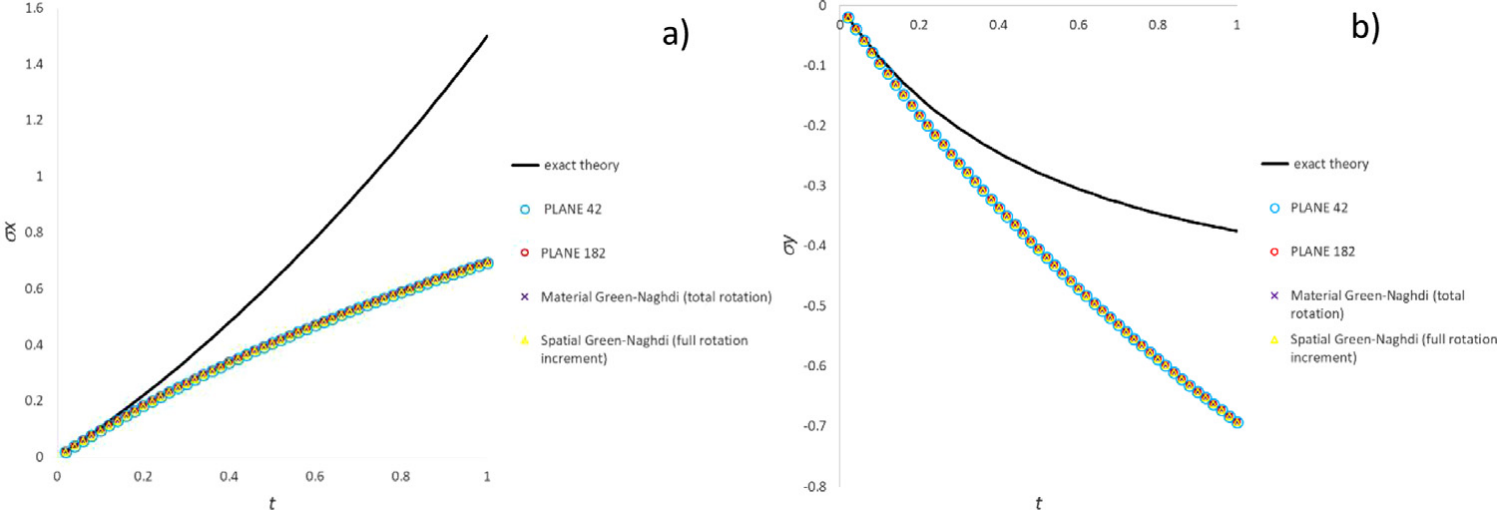
Stress history predicted by Ansys software and variants of CC algorithms for extension-compression test: a) σ_x_ component; b) σ_y_ component.

**Fig. 11:**
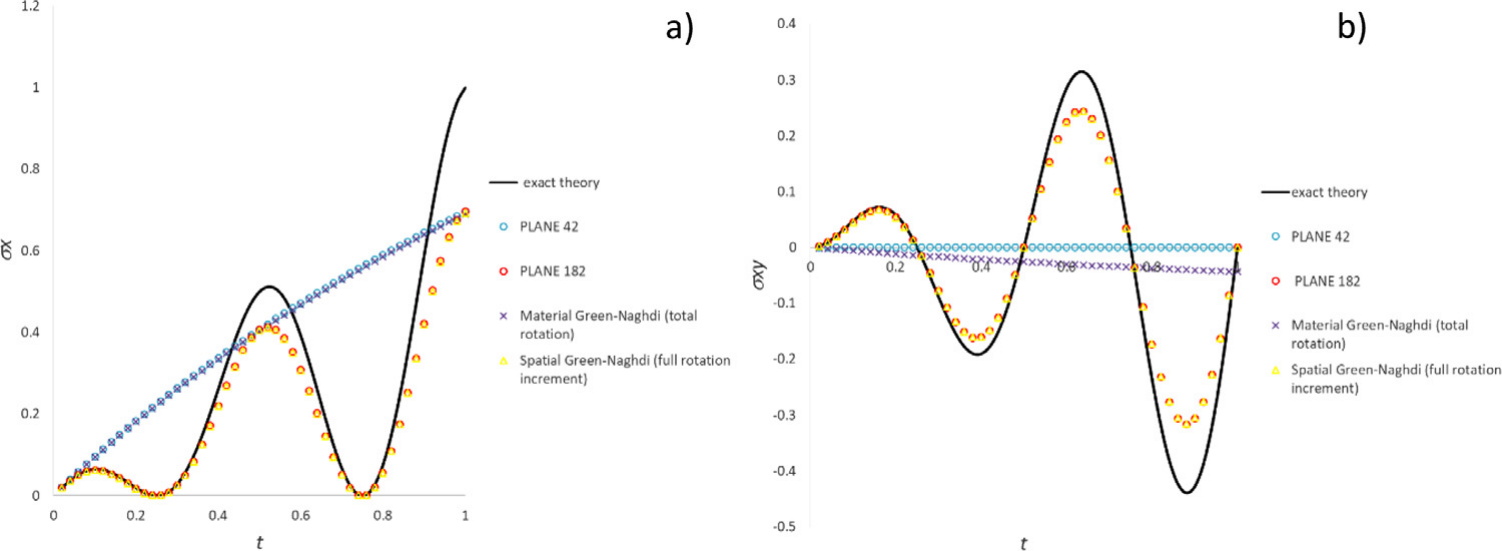
Stress history predicted by Ansys software and variants of CC algorithms for extension-rotation test: a) σ_x_ component; b) σ_xy_ component.

**Fig. 12:**
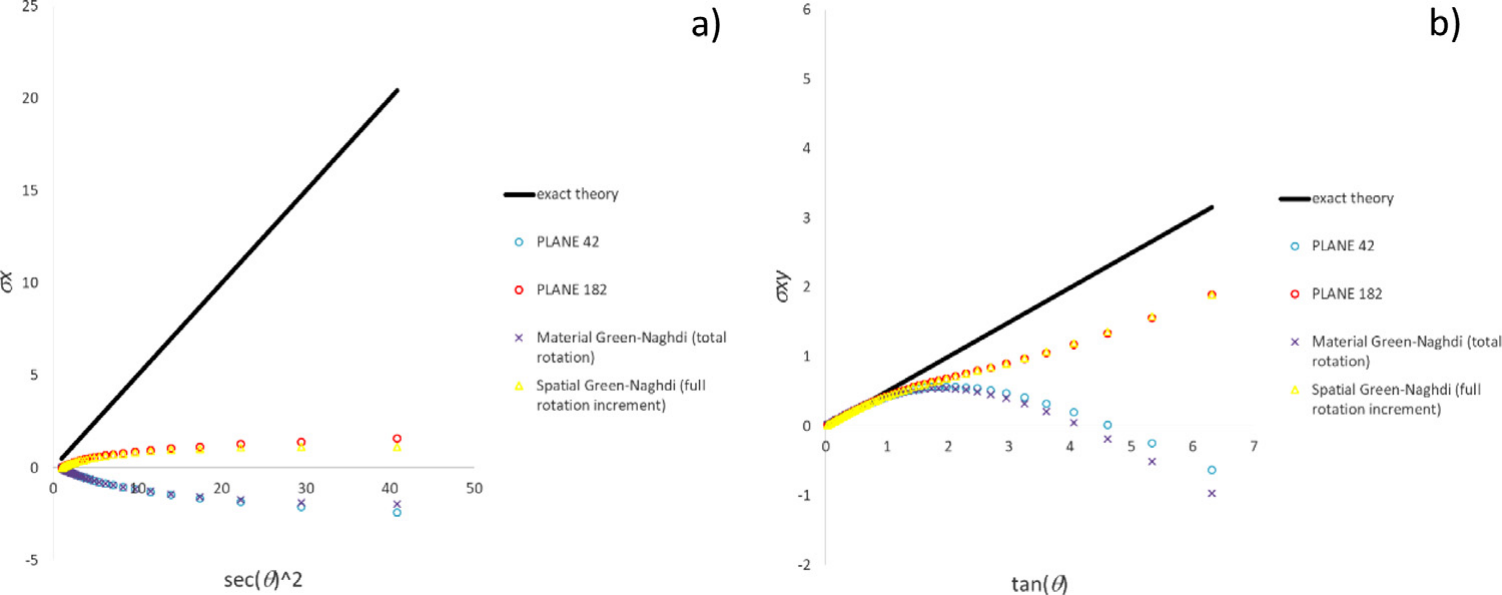
Stress history predicted by Ansys software and variants of CC algorithms for simple shear test: a) σ_x_ component; b) σ_xy_ component.

Fig. 10, Fig. 11, Fig. 12 plot for the three test cases including the most common particle motions the data (histories of the pertinent stress components) computed by means of Ansys software and the methods developed for commercial software emulation, i.e., “material Green-Naghdi (total rotation)” and “spatial Green-Nagdi (full rotation increment)”, which are summarized in the dedicated paragraph.

## Limitations

None.

## Ethics Statement

The authors have read and follow the ethical requirements for publication in Data in Brief. In this regard, the current work does not involve human subjects, animal experiments, or any data collected from social media platforms

## CRediT authorship contribution statement

**Fabrizio Antonio Stefani:** Conceptualization, Methodology, Software, Data curation, Writing – original draft. **Ramon Francesconi:** Conceptualization, Data curation, Validation, Writing – review & editing.

## Data Availability

Data for the Assessment of Stress Integration Schemes for Large-Deformation Finite Element Analysis (Original data) (Mendeley Data).
